# SARS-CoV-2 Genome Analysis of Japanese Travelers in Nile River Cruise

**DOI:** 10.3389/fmicb.2020.01316

**Published:** 2020-06-05

**Authors:** Tsuyoshi Sekizuka, Sanae Kuramoto, Eri Nariai, Masakatsu Taira, Yushi Hachisu, Akihiko Tokaji, Michiyo Shinohara, Tsuyoshi Kishimoto, Kentaro Itokawa, Yusuke Kobayashi, Keisuke Kadokura, Hajime Kamiya, Tamano Matsui, Motoi Suzuki, Makoto Kuroda

**Affiliations:** ^1^Pathogen Genomics Center, National Institute of Infectious Diseases, Tokyo, Japan; ^2^Ishikawa Prefectural Institute of Public Health and Environmental Science, Kanazawa, Japan; ^3^Chiba Prefectural Institute of Public Health, Chiba, Japan; ^4^Kochi Prefectural Institute of Public Health, Kochi, Japan; ^5^Saitama Prefectural Institute of Public Health, Saitama, Japan; ^6^Infectious Disease Surveillance Center, National Institute of Infectious Diseases, Tokyo, Japan

**Keywords:** cruise ship, imported case, genome epidemiology, single-nucleotide variations, haplotype network

## Abstract

Japan has reported 26 cases of coronavirus disease 2019 (COVID-19) linked to cruise tours on the River Nile in Egypt between March 5 and 15, 2020. Here, we characterized the severe acute respiratory syndrome coronavirus 2 (SARS-CoV-2) genome of isolates from 10 travelers who returned from Egypt and from patients possibly associated with these travelers. We performed haplotype network analysis of SARS-CoV-2 isolates using genome-wide single-nucleotide variations. Our analysis identified two potential Egypt-related clusters from these imported cases, and these clusters were related to globally detected viruses in different countries.

## Introduction

The current pandemic of coronavirus disease 2019^1^ (COVID-19) is caused by a positive-sense RNA virus, named the severe acute respiratory syndrome coronavirus 2 (SARS-CoV-2) (Coronaviridae Study Group of the International Committee on Taxonomy of V., 2020). As of April 6, 2020, Japan has confirmed 3,985 cases in total, excluding the cases in Diamond Princess cruise ship. This makes Japan one of the developed countries least affected by SARS-CoV-2. The Japanese government has focused on the identification and mitigation of emerging COVID-19 clusters before further expansion, a strategy considered optimal in a low infection rate situation. Japan has sustained moderate spread by focusing on COVID-19 outbreak clusters; however, an ever-increasing number of COVID-19 cases has made it difficult to identify all infection routes.

From the beginning of March 2020, 46 travelers who returned to Japan from abroad were suspected to have imported COVID-19; these cases account for roughly 10% of all new cases recorded in Japan. Among these imported cases, as many as 26 have been linked to cruise tours on the River Nile in Egypt between March 5 and 15, 2020. Most of the travelers visited Egypt from late February to early March and embarked on Nile River cruise ship tours between Cairo and Luxor for 3–4 days. Soon after they returned to Japan, they experienced the onset of fever and sore throat. They visited their respective local consultation centers for recent arrivals from abroad and underwent PCR testing, which confirmed them as being positive for SARS-CoV-2. A field epidemiological study was conducted on the people closely associated with them, such as family members, who might have been exposed to the virus. In this study, we have evaluated viral genome sequences from SARS-CoV-2-positive travelers who returned from Egypt, and characterized the haplotype networks to demonstrate possible routes of the spread.

## Results

**Figure 1:**
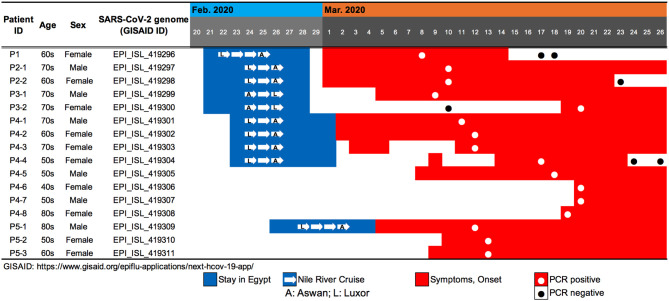
Summary of travel history, clinical course, and PCR testing for 10 SARS-CoV-2-positive travelers who returned to Japan from Egypt, as well as the associated patients who were their close contacts.

**Figure 2:**
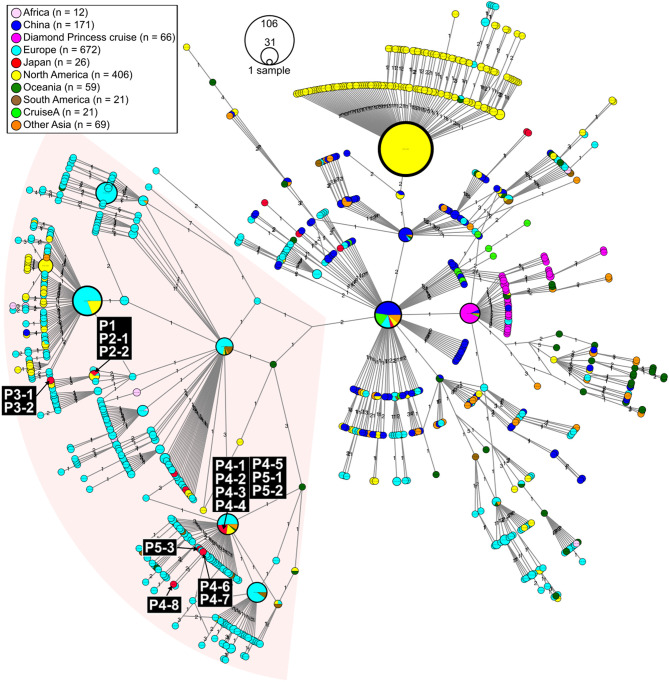
Haplotype network using genome-wide single-nucleotide variations (HN-GSNVs) of SARS-CoV-2 isolates in the world. Whole-genome sequences of SARS-CoV-2 isolates from 10 travelers who returned from Egypt and possible patients linked to them (Figure 1) were compared with all GISAID-available SARS-CoV-2 genomes (*n* = 1,507, updated on March 30, 2020. See Table S1) by median-joining SNV network analysis. The numbers on the edges indicate differential SNVs between pair-wise nodes (isolates). SARS-CoV-2 disseminated from the end of December, 2019, from Wuhan City in China, one of the potential origins of Wuhan-Hu-1, isolated on December 26, 2020 (GenBank ID: MN908947). Wuhan-Hu-1 is plotted at the center of the haplotype network. Currently, at least three clades have disseminated globally in a region-specific manner.

We evaluated viral genome sequences from 10 SARS-CoV-2-positive travelers who returned from Egypt, as well as their close contacts, to identify possible routes of spread. The travel histories, clinical courses, and PCR testing results are summarized in Figure 1. To characterize the potential origins and routes of the suspected imported cases, we determined the whole-genome sequence of SARS-CoV-2 using a multiplex PCR-based RNA-Seq by ARTIC Network protocol^2^ based on PrimalSeq (Quick et al., 2017; Grubaugh et al., 2019) with modified primers and protocol (Itokawa et al., 2020). For the obtained genome sequences, haplotype network analysis using genome-wide single-nucleotide variations (SNVs) on the core regions from positions 99 to 29,796 nt in the Wuhan-Hu-1 reference genome sequence (GISAID ID, EPI_ISL_402125; GenBank ID, MN908947.3) was performed (Figure 2). SARS-CoV-2 genome sequences with nearly full-length information (≥ 29 kb) were retrieved from the GISAID EpiCoV database on March 30, 2020, and we generated haplotype networks by median-joining network analysis using PopART software^3^ to highlight and trace a potential infectious route among COVID-19 patient populations.

**Figure 3:**
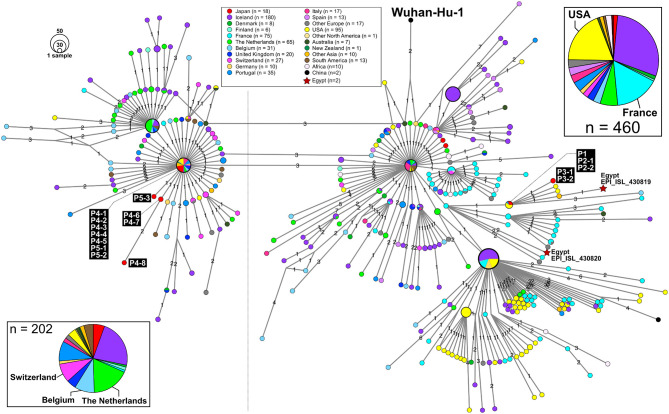
An excerpt of HN-GSNVs from Japanese travelers returning from Egypt and patients associated with them. The haplotype of SARS-CoV-2 genome sequences for 16 patients (Figure 1) was found in the two marked clusters, which comprised the most European isolates (Figure 2). See the legends in Figure 2 for details.

Patient P1 (P1; hereafter, patients are designated in this manner) arrived at Cairo airport from Tokyo on February 22 and embarked on a Nile River cruise ship for 4 days (Figure 1). The SARS-CoV-2 genome sequence of the P1 isolate shows a close lineage with European isolates, with several SNVs (Figure 3). P2-1 and P2-2 had visited Egypt together and traveled aboard the same Nile River cruise ship, and SARS-CoV-2 genome sequences isolated from them are identical with that of P1 (Figure 3).

The couple P3-1 and P3-2 visited Egypt together, on the same Tokyo to Cairo flight as the above P1 patient but boarded a different Nile River cruise ship. The husband, P3-1, showed flu-like symptoms on March 5 and was confirmed with COVID-19 on March 9, while the wife, P3-2, was asymptomatic and PCR negative on March 10 despite their close contact during the trip. 10 days later, however, on March 20, P3-2 exhibited symptoms and was confirmed with COVID-19. These two SARS-CoV-2 isolates show identical genome sequence (Figure 3) and are distinct from the genome sequences (P1, P2-1, and P2-2) by only one SNV (Figure 3).

Meanwhile, compared to the genome sequences of the above five patients, the SARS-CoV-2 genome sequence obtained from following P4 and P5 patients showed clearly different haplotype lineage, with at least five differential SNVs (Figure 3). Four patients (P4-1 to P4-4) had visited Egypt at the end of February, a few days after the above-mentioned patients, and exhibited symptoms after returning back to Japan. Intriguingly, the genome sequences of four additional patients (P4-5–P4-8), having no history of recent overseas travel or contact with the above patients, were markedly close to the P4-related isolates. P4-6 and P4-7 are coworkers with P4-5, and P4-8 is mother of P4-5, indicating that three patients (P4-6–P4-8) were close contacts to P4-5 as original source. This finding demonstrated the identification of a potential hidden link to the import of infection from Egypt.

P5-1 had visited Egypt and embarked on a Nile River cruise ship different from the other travelers mentioned above. He showed symptoms and tested positive by PCR after returning home. His relatives, P5-2 (daughter) and P5-3 (sister), who had close contact with him, subsequently showed symptoms and tested positive for SARS-CoV-2 by PCR (Figure 1). The SARS-CoV-2 genome sequence of P5-1 was identical to that of P5-2, distinct only by one SNV from that of P5-3, indicating direct infections from P5-1 to P5-2 and P5-3, (Figure 3).

Thus far, two genome sequences of SARS-CoV-2 isolates in Egypt (isolation date: 2020/03/18; GISAID ID: EPI_ISL_430819 and EPI_ISL430820) are available in GISAID, and the haplotype network exhibits that P1 and P3 patients are closely related to those Egypt isolates with 2 or 3 SNVs (Figure 3).

## Discussion

In this study, we found two SARS-CoV-2 genome lineages from Egypt-related imported cases. These virus lineages belonged to a single clade rooted to the major indexed isolates, which diverged from Wuhan-Hu-1 by several clades. The members in this clade are considered to be circulating in multiple countries, mainly in the Europe and South America. The Egypt-related isolates described in this study are divided into two distinct SARS-CoV-2 haplotype lineages, with two or three additional SNVs from the major indexed isolates; one lineage, including P1–P3, included most haplotypes isolated from France and Egypt (Figure 3), whereas the other lineage, including P4 and P5, included the Netherlands/Belgium/Switzerland isolates.

On 6 March, the Egyptian Health Ministry confirmed 12 COVID-19 cases among the Egyptian crew staff aboard a Nile River cruise ship. On 7 March, the Egyptian health authorities announced that 45 people on board that ship had tested positive, and that the ship had been subjected to quarantine at a dock in Luxor. It is also speculated that Egypt probably has a large burden of COVID-19 cases that are unreported, and Egypt might be a source of COVID-19 export that is not yet accounted for by many public health initiatives (Tuite et al., 2020). Since early March this year, the number of reported COVID-19 cases has been rapidly increasing in Europe countries. This study suggested that patients with a history of travel to Egypt and embarking on Nile River cruises between mid-February and early March could be one of the potential sources of COVID-19 cases imported into Japan.

## Materials and Methods

### Clinical Specimens and RT-qPCR Testing for COVID-19

Pharyngeal specimens were collected from patients, and a quantitative reverse transcription PCR (RT-qPCR) testing for SARS-CoV-2 (Jung et al., 2020; Shirato et al., 2020) was performed.

### Whole Genome Sequencing of SARS-CoV-2

Basically, whole genome sequences of SARS-CoV-2 was obtained by PrimalSeq protocol to enrich cDNA of SARS-CoV-2 genome by multiplex RT-PCR amplicons using a multiplexed PCR primer set which was proposed by Wellcome Trust ARTIC Network. We found particular two amplicons regularly showed low to zero coverage due to primer dimerization as described in Itokawa et al. (2020), we used the modified primer for the multiplex PCR amplifications (Itokawa et al., 2020). The PCR products from same clinical sample was pooled, purified and subjected for Illumina library construction using QIAseq FX DNA Library Kit (QIAGEN, Hilden Germany). NextSeq 500 platform (Illumina, San Diego, USA) was used for sequencing the indexed libraries. The NGS reads were mapped to the SARS-CoV-2 Wuhan-Hu-1 reference genome sequence (29.9 kb ss-RNA; GenBank ID: MN908947), resulting to the specimen-specific SARS-CoV-2 genome sequence by fully mapping on the reference. These mapped reads of SARS-CoV-2 sequences were assembled using A5-miseq v.20140604 (Coil et al., 2015) to determine the full genome sequence (see the details in Table S1). The SNV sites and marked heterogeneity were extracted by the read-mapping at ≥10 × depth and from 99 to 29,796 nt region of Wuhan-Hu-1 genome sequence (see the details in Table S2).

### Comparative Genome Sequence Analysis and Single Nucleotide Variation Analysis

The nearly full-length genome sequence (≥ 29 kb) of SARS-CoV-2 were retrieved from GISAID EpiCoV database in March 10, 2020, followed by multiple alignment using MAFFT v7.222 (Katoh and Standley, 2013). The poorly aligned regions in 5′ and 3′ end were trimmed; we determined that the core regions were from 99 to 29,796 nt position against Wuhan-Hu-1 genome sequences (GISAID ID, EPI_ISL_402125; GenBank ID, MN908947.3). Gap-containing sequences in the core region were excluded; sequences of 1,507 isolates in GISAID database were eventually used in subsequent analyses (updated on March 30, 2020. See Table S1). The genome sequences were aligned using MAFFT program together with sequences retrieved from database, followed by extraction of SNV and deletion sites. The SNV median-joining network analysis was performed by PopART software^3^.

## Data Availability Statement

The new sequences have been deposited in GISAID with accession IDs EPI_ISL_419296 – EPI_ISL_419311, in addition, in DDBJ/NCBI/EBI with accession IDs LC547518 – LC547533.

## Ethics Statement

The studies involving human participants were reviewed and approved by the National Institute of Infectious Diseases in Japan (Approval No. 1091). It was conducted according to the principles of the Declaration of Helsinki, in compliance with the Law Concerning the Prevention of Infections and Medical Care for Patients of Infections of Japan. The ethical committee waived the need for written consent regarding the research into the viral genome sequence. The personal data related to the clinical information were anonymized, and our procedure is not to request written consent for all patients suffering from COVID-19. Written informed consent for participation was not required for this study in accordance with the national legislation and the institutional requirements.

## Author Contributions

TS, KI, and MK designed and organized the genome study. SK, EN, MT, YH, AT, MSh, and TK performed the laboratory detection. TS and KI performed the genome analysis. YK, KK, HK, TM, and MSu contributed to the field epidemiological study. MK wrote the manuscript.

## Conflict of Interest

The authors declare that the research was conducted in the absence of any commercial or financial relationships that could be construed as a potential conflict of interest.

## Footnotes

^1^*Coronavirus Disease 2019 (COVID-19) Pandemic: Increased Transmission in the EU/EEA and the UK–Seventh*. Available online at: https://www.ecdc.europa.eu/sites/default/files/documents/RRA-seventh-update-Outbreak-of-coronavirus-disease-COVID-19.pdf (accessed March 25, 2020).

^2^ARTIC Network protocol. Available online at: https://artic.network/ncov-2019.

^3^PopART_software: http://popart.otago.ac.nz.
